# Simulations of surface charge density changes during the untreated solid tumour growth

**DOI:** 10.1098/rsos.220552

**Published:** 2022-11-30

**Authors:** Henry Bory Prevez, Argenis Adrian Soutelo Jimenez, Eduardo José Roca Oria, José Alejandro Heredia Kindelán, Maraelys Morales González, Narciso Antonio Villar Goris, Nibaldo Hernández Mesa, Victoriano Gustavo Sierra González, Yenia Infantes Frometa, Juan Ignacio Montijano, Luis Enrique Bergues Cabrales

**Affiliations:** ^1^ Departamento de Control Automático, Facultad de Ingeniería Eléctrica, Universidad de Oriente, Santiago de Cuba, Cuba; ^2^ Departamento de Química, Facultad de Ciencias Naturales y Exactas, Universidad de Oriente, Santiago de Cuba, Cuba; ^3^ Departamento de Física, Facultad de Ciencias Naturales y Exactas, Universidad de Oriente, Santiago de Cuba, Cuba; ^4^ Departamento de Farmacia, Facultad de Ciencias Naturales y Exactas, Universidad de Oriente, Santiago de Cuba, Cuba; ^5^ Departamento de Ciencia e Innovación, Centro Nacional de Electromagnetismo Aplicado, Universidad de Oriente, Santiago de Cuba, Cuba; ^6^ Facultad de Ciencias Sociales, Universidad de Oriente, Santiago de Cuba, Cuba; ^7^ Universidad Autónoma de Santo Domingo, Santo Domingo, República Dominicana; ^8^ Centro de Neurociencias de Cuba (CNEURO), La Habana, Cuba; ^9^ Grupo de las Industrias Biotecnológica y Farmacéuticas (BioCubaFarma), La Habana, Cuba; ^10^ Departamento de Matemática Aplicada, Instituto Universitario de Matemática y Aplicaciones, Universidad de Zaragoza, Zaragoza, España

**Keywords:** surface charge density, Gompertz equation, electrical conductivity, electrical permittivity, unperturbed solid tumour growth kinetic

## Abstract

Understanding untreated tumour growth kinetics and its intrinsic behaviour is interesting and intriguing. The aim of this study is to propose an approximate analytical expression that allows us to simulate changes in surface charge density at the cancer-surrounding healthy tissue interface during the untreated solid tumour growth. For this, the Gompertz and Poisson equations are used. Simulations reveal that the unperturbed solid tumour growth is closely related to changes in the surface charge density over time between the tumour and the surrounding healthy tissue. Furthermore, the unperturbed solid tumour growth is governed by temporal changes in this surface charge density. It is concluded that results corroborate the correspondence between the electrical and physiological parameters in the untreated cancer, which may have an essential role in its growth, progression, metastasis and protection against immune system attack and anti-cancer therapies. In addition, the knowledge of surface charge density changes at the cancer-surrounding healthy tissue interface may be relevant when redesigning the molecules in chemotherapy and immunotherapy taking into account their polarities. This can also be true in the design of completely novel therapies.

## 1. Introduction

The untreated solid tumour growth kinetics (TGK) exhibits a sigmoidal shape and its understanding constitutes a challenge for researchers [1,2]. Several equations are used to describe TGK, such as: the conventional Gompertz (CGE, the most accepted) [1,3], Montijano-Bergues-Bory-Gompertz [4], modified Kolmogorov-Johnson-Mehl-Avrami, Logistic & Bertalanffy [1]. The parameters of the first three equations are interconnected [5]. These equations involve different biological parameters (i.e. intrinsic growth rate, endogenous anti-angiogenesis, carrying capacity of the tumour) obtained from fitting experimental data (tumour mass and volume, and cancer cells number). Nevertheless, none of these equations include bioelectrical parameters related to electrical properties and the active bioelectricity (or bioelectric potential) of the cancer, surrounding healthy tissue, and interface between these two tissues, named *Σ*.

The electrical properties and active bioelectricity inherent in cancer and surrounding healthy tissue are experimentally confirmed by means of several techniques, such as: bioelectrical impedance analysis [6], image technique of the electric current density [7], microelectrodes and neutralized input capacity amplifiers, high-impedance micropipettes, potentiometry, fluorescence, electrical double layer in field-effect transistors, electrical impedance spectroscopy together with other devices [8–14]. Additionally, vibrating probes, glass microelectrodes, microfluidic-based tissue/organ-on-a-chip devices, and endoscopes with inserted electronics to detect bioelectricity changes in real-time are recommended. Nanotechnology-based bioelectronics with nano-sized devices is used to quickly detect cancer at an earlier stage [8,9]. The bioelectricity-driven nanoparticle binding is suggested instead of static electrical potential via electrophoresis. The bioelectricity is proposed to capture electrostatically and magnetically circulating cancer cells from the entire blood to investigate their metabolic state [15].

Several findings have been revealed, such as: (1) differences between electrical conductivities (*η_k_*, *k* = 1,2) and electrical permittivities (*ε_k_*, *k* = 1,2) of the untreated malignant tumour (*k* = 1) and the surrounding healthy tissue (*k* = 2) [11]; (2) these two physical properties as a potential diagnostic method [16]; (3) differences between ionic current (due to the movement of charged ions) and faradic current (produced by electrons exchange from reduction and/or oxidation of biochemical molecules) in cancer and surrounding healthy tissue [8]; (4) the existence of chemical and electrical (charged negatively) environments in cancer cells and untreated tumours [1,4,14] and their key roles in the genesis, growth, progression, metastasis and treatment of cancer [17]; (5) the impact of tumour microenvironment on its electrical properties [16]; (6) the breakdown of intercellular communication (gap junction) in the tumour due to low regulation in expression of the connexin [12,18]; (7) negative electrical biopotentials in the tumour and positive electrical biopotentials in the surrounding healthy tissue [12–14]; (8) cancer cells and some cells of the immune system negatively charged [14]; (9) weaker electrical coupling among cancer cells and the association of deregulation of intercellular communication with tumourigenicity and metastasis of the cancer [14,18]; (10) bioelectronic cancer regulator as an initiator of the mitosis and deoxyribonucleic acid synthesis; and (11) correction of alterations in the electrical communication system of cancer by manipulating its bioelectrical properties, known as bioelectronic medicine [8].

The uneven movement of ions and electrons across the plasma membrane via ion pumps modifies the imbalance of the charge between the intra- and extracellular compartments. This ionic imbalance, gene expression level, glutamate-dependent currents and both ionic and faradic currents explain the active cancer bioelectricity [8]. Furthermore, the ionic imbalance on both sides of the cancer cell membrane involved in the deregulation of ionic activity (a novel hallmark of cancer cells), altered membrane electrical potential difference (*V*_mem_), shape change, pH, heterogeneity, phenotype, metabolism abnormalities, growth signalling, proliferation, tumourigenesis, angiogenesis, invasion and metastasis of the cancer cells, as well as in the plasticity, heterogeneity and cellular networks of cancer [8,16,19–22]. The intra-tumour heterogeneity and anisotropy have an essential role in its growth, metastasis and resistance to anti-cancer therapies [1,2,4]. The cancer phenotypes include both cellular ionic and faradic currents. The tumour growth may be due to malfunctions in the bioelectrical circuitry of their cells. The tumour progression may be explained by the alterations of trans-plasma membrane electron transport. And the tumour metastasis considers the degradation of basement membranes, cancer cell invasion, migration, extravasation and colonization [8].

Biological processes form bioelectric circuits from individual cell behaviours and anatomical information encoded in bioelectrical states to achieve a better control over spatio-temporal biological patterns. Electrically active cancer cells possess bioelectric circuitry that generates resting membrane potential and endogenous electric fields that influence cell functions and communication [8,23]. Endogenous electric potential gradients (established across multiple cells due to gap junctions and other cell-to-cell connections on a tissue level) induce small endogenous electric fields, which are responsible for altered migration and invasiveness of cancer cells [18,24].

Alterations in *V*_mem_ are involved in high proliferation (due to the depolarization of their membranes by higher intracellular concentration of sodium ions) and mitosis, depletion of adenosine triphosphate, failure of ionic pumps at the cellular membrane and mechanism of contact inhibition of cancer cells. A depolarized membrane is considered a driving force for the production of Ca^2+^ and bioelectronic cancer regulator that affect proliferation, migration, invasion and metastasis of cancer cells [8,12–14]. Furthermore, changes in *V*_mem_ are related to the modulation of local concentrations of signalling molecules and ions, the spatio-temporal regulation of morphogenesis, the interaction with heterogeneous networks (that combines conventional gene regulatory network) is controlled by spatio-temporal bioelectrical patterns based on electric potentials and currents from steady and oscillatory multicellular states, among others. In turn, these spatio-temporal bioelectrical patterns influence the spatiotemporal distributions of signalling ions and molecules that modulate biochemical pathways in cancer cells, and therefore in growth and regeneration [8,9,25].

*V*_mem_ may be regulated in different ways, such as: the ion channel expression, the ionic composition of the extracellular environment, and the presence of bioelectronic gradients within cancer [8,20]. Therefore, Payne *et al*. [20] suggest that *V*_mem_ should be analysed in two directions: *V*_mem_ effect on the cellular function (that contributes to the cancer phenotype) and how *V*_mem_ is affected (by the expression of voltage-gated ion channels and cell metabolism). Alterations in the metabolism of cancer cells modify *V*_mem_ [8,15,26]. Bioelectrical pathways associated with a metabolic phenomenon affect ionic electrical-based communication among cancer cells, like: reactive oxygen species and aberrant trans plasma membrane electron transport systems. The reverse Warburg effect is induced in cancer cells by higher levels of reactive oxygen species, which may be caused by malfunction in the redox balance, altered biological electron transfer reactions (higher electron transfer), a high energetic demand, increased concentration of reduced bioelectrochemical mediators, and participation of the trans plasma membrane electron transport systems in oxidation; and redox centres existing in cell membranes transport electrons across these membranes in the form of faradic currents [8,26].

The above-mentioned results corroborate the close relationship between biological and electrical parameters in tissues. Likewise, it confirms that the bioelectrical activity in all cell types (e.g. cancer) is involved in many physiological mechanisms. Nevertheless, bioelectrical pathways are still poorly understood in cancer cells, TGK and *Σ*. This should be taken into consideration because both tissue types have different electrical properties and bioelectrical activities. Therefore, understanding of the bioelectricity in cancer and surrounding healthy tissue constitutes a challenge for researchers.

It is documented in electrodynamics of media that a surface charge density arises at the interface between two materials in contact with different electrical properties [27]. Therefore, a surface charge density (*σ*_12_) is expected at *Σ* for the following reasons: firstly, solid tumours have chemical and electrical environments [1,4,14]. Secondly, the cancer and its surrounding healthy tissue are in contact and heterogeneous [28]. Thirdly, both tissue types differ significantly in their electrical properties and thermal [10,13,14,29,30] and physiological parameters [8,14]. Fourthly, *σ*_12_ is due to synergism between an external volumetric current density (the source of electricity) and the Maxwell-Wagner-Sillars interfacial polarization. The Maxwell-Wagner-Sillars effect is an interfacial relaxation process that occurs for all two-phase multi-systems, in which the electric current must pass an interface between two different loss dielectrics [28,31,32]. Lastly, the electrophysiological activity in cancer (in tumour regions near *Σ* mainly) is higher than that in the surrounding healthy tissue [8,9,14,33].

*σ*_12_ has been measured in many biological and non-biological heterogeneous materials by means of the surface photovoltage effect, the vibrating probe technique, electrostatic force microscopy, among others [34,35]. Nevertheless, in the literature *σ*_12_ at *Σ* has not been experimentally measured nor calculated theoretically in cancer. Estimation of *σ*_12_ at *Σ* presupposes the experimental knowledge of normal components of the flux density vector on both sides of *Σ*, a procedure that is cumbersome and expensive (in time and resources) in preclinical and clinical studies. Furthermore, an analysis of TGK in terms of *η_k_*, *ε_k_* (*k* = 1,2) and *σ*_12_ has not been reported in the literature. *σ*_12_ is ignored and cannot be estimated from the vast experimental data available. The aforementioned are the aspects we mainly take into consideration for using the physic-mathematical modelling in our research. Therefore, the aim of this study is to propose an approximate analytical expression that allows us to simulate *σ*_12_ at *Σ* during the untreated tumour growth, in terms of two tumour kinetic parameters, tumour radius and electrical properties of the tumour and its surrounding healthy tissue.

## 2. Methods

### 2.1. Assumptions

**Figure 1.**
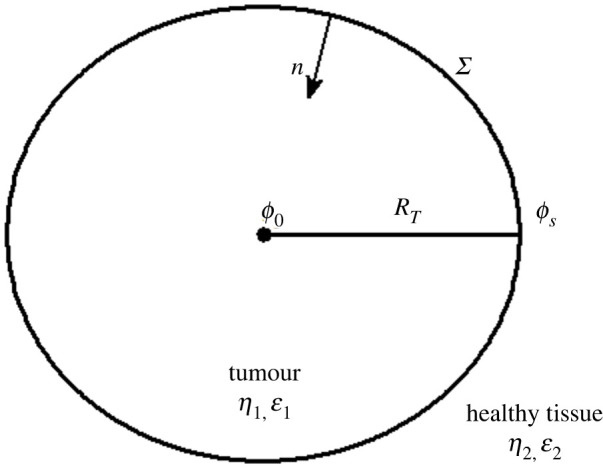
Schematic representation of a spherical tumour surrounded by its healthy tissue. Variables *ϕ*_0_ and *ϕ*_s_ denote the electrical potentials in the centre and the periphery of the tumour, respectively. *R_T_*_0_ is the initial tumour radius. *η*_i_ and *ε*_i_ represent the electrical conductivities and electrical permittivities of the tumuor (*i* = 1) and the surrounding healthy tissue (*i* = 2). *n* denotes the inward unit normal vector to the boundary *Σ* (interface that delimits both tissues).

1. There is a three-dimensional, conductive, anisotropic and heterogeneous region consisting of two linear, anisotropic and heterogeneous media (tumour and the surrounding healthy tissue) separated by an interface *Σ* (figure 1). Untreated solid tumour (medium inside *Σ*, named medium 1) is considered as a heterogeneous conducting sphere of radius *R*_T_ (in m) of constant mean conductivity (*η*_1_, in *S*/*m*) and mean permittivity (*ε*_1_, in *F*/*m*). The surrounding healthy tissue (medium outside *Σ*, named medium 2) is supposed to be a heterogeneous infinite medium of constant mean conductivity (*η*_2_, in *S*/*m*) and mean permittivity (*ε*_2_, in *F*/*m*), where *η*_1_ > *η*_2_ and *ε*_1_ > *ε*_2_.
2. The source of electricity is neglected because the tumour is unperturbed.
3. Maxwell-Wagner-Sillars effect occurs physiologically between the tumour and the surrounding healthy tissue (see Introduction section).
4. In a first approximation, the electromotive force field (***E***_ *f*_) depends only on the distance to the tumour centre.
5. Normal and cancer cells that are at *Σ* do not significantly contribute to (***E***_ *f*_).

### 2.2. Further comments about assumptions 1, 4 and 5

The first assumption may be approached from the physical point of view. As solid tumour and surrounding healthy tissue are anisotropic and heterogeneous media (formed by cells, water, ions, molecules, macromolecules, among others) [10,29,30], we consider that $\overset{↔}{\eta }$ and $\overset{↔}{\varepsilon }$ are real symmetric second-order tensors (3 × 3 matrix symmetric) of electrical conductivity and electrical permittivity, respectively. When the electrical conductivity and electrical permittivity are referred to the principal axes and both the electric field and the current density are related to the same coordinate system, then all nondiagonal elements are equal to zero and this 3 × 3 symmetric matrix becomes diagonal. Consequently, there is an orthonormal base (which defines the so-called principal axes of the medium) in which $\overset{↔}{\eta }$ is represented by the diagonal matrix diag(*η*_1_,*η*_2_,*η*_3_), where *η*_1_, *η*_2_ and *η*_3_ are electrical conductivities according to these main axes. If these diagonal elements are replaced by their mean value, named *η* (*η* = (*η*_1_ + *η*_2_ + *η*_3_)/3) in this approximation, the tensor $\overset{↔}{\eta }$ corresponds to the scalar matrix *η*I, where I is the identity matrix of order 3, as in [30]. The tensor $\overset{↔}{\varepsilon }$ is treated in the same manner and its mean value is *ε*.

Although cancer and surrounding healthy tissue are heterogeneous and anisotropic media, most experimental studies report their respective average values of $\overset{↔}{\eta }$ and $\overset{↔}{\varepsilon }$ tensors [6,10–14,28,29,36,37]. Furthermore, *η*_1_ > *η*_2_ and *ε*_1_ > *ε*_2_ have been explained because malignant tumours have a significantly higher water content, higher concentrations of ions and electrons, and altered cellular metabolism compared to those in the surrounding healthy tissue [6,8–10,15,16,19,38].

Tumour borders play a crucial role in growth, metastasis, aggressiveness and anti-cancer therapy planning [1,4,38]. Locating them is not easy from a clinical point of view because the tumour border is a marginal zone that contains tumour cells and normal cells [39]. In this study, interface *Σ* is the tumour-free margin, according to pathological anatomy reports. This ensures that there is no infiltration of the tumour into the surrounding healthy tissue and there are two well-defined regions instead. In addition, the geometry and border of the tumour (regular or irregular) have no relevance for this tumour-free margin; therefore, the tumour contour may be assumed regular, sharp and smooth. It should be noted that *Σ* is not chosen as the surgical margin because it does not guarantee that the tumour has infiltrated adjacent normal tissue [39].

The term ‘infinite healthy tissue’ does not mean an unlimited space but rather refers to an enormous healthy tissue (the limited region free of infiltration and metastasis of cancer cells) in comparison to the tumour.

A standard pattern of three-dimensional anatomically realistic models (numerically solved with COMSOL-Multiphysics and similar packages) is very unlikely to be established in simulations because it requires precise knowledge of the electric properties (i.e. electrical conductivity, electrical permittivity) and physiologic characteristics (i.e. type, heterogeneity, anisotropy, size, shape, composition, structure, consistency and water content) of both tissues. This becomes even more cumbersome when other characteristics of the tumour are taken into account, such as: histological variety, stage, stiffness, mitotic index, degree of anaplasia, invasiveness and metastasis. In addition, our vast experience in preclinical studies has shown differences in space–time patterns of a same tumour histological variety that grows in several BALB/c/Cenp mice under the same experimental conditions (temperature and relative humidity of the room; initial concentration and viability of cancer cells; and mice with the same age, gender and weight) [1,2,4]. This result is due to the biological individuality. If this analysis is individualized, an individual model should be suggested for each patient/animal, which is not feasible from a theoretical and experimental standpoint. Furthermore, the diversity and complexity of non-spherical geometries [1–4] and irregular borders [7,10,38,40] of tumours during their growths makes it very difficult to establish a single spatio-temporal pattern of these two aspects for simulations. The electrical and biological parameters of the tumours cannot be controlled by the performing physician (in clinics) or researchers (*in vitro* and *in vivo* studies). That is why tissue realistic conditions are not considered in this study.

The tumour spherical geometry is observed in three-dimensional culture [41,42] and first time instants of TGK (tumour sizes ≤ 50 mm^3^) [1,2,4]. Spherical cancer models (major three-dimensional *in vitro* models) have been used in cancer research as an intermediate model between *in vitro* cancer cell line cultures and *in vivo* tumour. These models have gained popularity in screening environments for better assessment and characterization of anti-cancer therapy efficacy (i.e. chemoresistance, radioresistance), and identifying potential cancer therapeutics, among others applications. Chemoresistance and radioresistance of cancer may be more marked in spherical tumours than those in non-spherical tumours, according to the results of the simulations reported by Castañeda *et al*. [43], and the sphere is the only geometry that is in contact with another surface at a point. Furthermore, they can be used as reliable models of *in vivo* solid tumours and drug screening platforms. Tumour spheroids may contribute to decreased animal experimentation [41,42]. The aspects and the poor understanding of *σ*_12_ at *Σ* from both experimental and theoretical points of view (unknown effects of the irregular border and changes in *σ*_12_ at *Σ*) are the reasons why we use the tumour spherical shape and symmetry to know *σ*_12_ at *Σ* approximately.

Until now, space–time distributions of ***E***_ *f*_ are neither experimentally nor theoretically known. In this study, ***E***_ *f*_ represents the active bioelectricity of unperturbed cancer and due to endogenous electrical biopotentials (*ϕ*) and/or intrinsic electrical sources in it [8,9,14–16,19–26]. Miklavčič *et al*. [44] measure experimentally *ϕ* along axial (*z*-axis) and radial (*r*-axis) directions in two tumour types (LLC and fibrosarcoma Sa-1). They report several findings, such as: *ϕ* is negative in the entire tumour; *ϕ* is more electronegative in the tumour centre (−160 mV for LLC tumour and −131.5 mV for fibrosarcoma Sa-1); electronegative of *ϕ* is less negative towards the periphery in LLC and fibrosarcoma Sa-1 tumours; and values of *ϕ* depend on distance (from tumour centre to its periphery) and not on angular coordinates. It is important to point out that *ϕ* should not be confused with the electric potential applied to a tissue by means of electrodes [2,30]. These are the reasons why the above-mentioned fourth assumption is proposed.

Although the tumour border is a marginal zone that contains tumour cells and normal cells from a clinical point of view, cancer cells at *Σ* invade the surrounding healthy tissue and do not migrate to the tumour interior. As a result, these cells do not contribute to ***E***_ *f*_. As ***E***_ *f*_ is only related to the unperturbed cancer active bioelectricity, normal cells at *Σ* do not contribute to ***E***_ *f*_. In addition, normal cells at *Σ* are replaced in cancer cells during tumour growth. These aspects justify the fifth assumption.

### 2.3. Theory

The assumptions in §2.1 and the close relationship between physical and biological aspects in cancer allow us to consider that *ϕ* and ***E***_ *f*_ are related, in a first approximation, by means of the equation

2.1 $$\nabla ∙\overset{↔}{\eta }∙(-\nabla \phi +E_{f})=0,$$

where $\overset{↔}{\eta }$ is the symmetric second-order tensor of the electrical conductivity of any linear, anisotropic and non-homogeneous medium (for example, a biological tissue). This tensor is used in previous studies [29,30].

Equation (2.1) is obtained by combining the continuity equation (∇∙J+∂ρ/∂t=0) for the static case (∂ρ/∂t=0) and law of Ohm $\left(J=\overset{↔}{\eta }∙(E+E_{f})\right)$, valid for media of linear conduction. In this case, J=J(r) is the electric current density J(r)=ρ(r)ν(r), where ρ(r) is the electric charge density and ν(r) the velocity field of electric current carriers.

Assumptions 2–5 in §2.1 allow us to consider that D=εE and the medium is considered isotropic in this approach, where ***D*** is the induction field (flux density vector). Taking this into account, and assuming that the medium is electrically homogeneous, equation (2.1) has the form

2.2 $$\nabla ∙\eta (-\nabla \phi +E_{f})=\eta \nabla ∙(-\nabla \phi +E_{f})=\eta [-\nabla^{2}\phi +\nabla ∙E_{f}]=0.$$

Therefore,

2.3 $$\nabla^{2}\phi =\nabla ∙E_{f}.$$

### 2.4. Boundary conditions

The region of interest is assumed as a heterogeneous biological tissue formed by the solid tumour (with average values *η*_1_ and *ε*_1_) surrounded by the surrounding healthy tissue (with average values *η*_2_ and *ε*_2_), as shown in figure 1.

According to the continuity equation for the static case, the current density normal components of the tumour (*J*_1*n*_) and the surrounding healthy tissue (*J*_2*n*_) are continuous at *Σ*

2.4 $$J_{1n}=J_{2n}.$$

Therefore,

2.5 $$\eta_{1}E_{1n}=\eta_{2}E_{2n}\Rightarrow \eta_{1}\frac{\partial \phi_{1}}{\partial n}=\eta_{2}\frac{\partial \phi_{2}}{\partial n},$$

where *E*_1*n*_ is the normal component of the electrical field of the tumour. *E*_2*n*_ is the normal component of the electrical field of the surrounding healthy tissue. *ϕ*_1_ is the electrical potential in the tumour and *ϕ*_2_ the electrical potential in the surrounding healthy tissue. The normal derivatives of *ϕ*_1_ and *ϕ*_2_ are $\partial \phi_{1}/\partial n$ and $\partial \phi_{2}/\partial n$, respectively.

Equation (2.4) is valid if $E_{f}=0$ at *Σ* (see Assumption 9). The positive normal to the tumour surface is indicated as a unit vector ***n*** (represented schematically in figure 1 by ***n***) drawn from the surrounding healthy tissue (medium 2) into the tumour (medium 1). According to this convention, medium 2 lay on the negative side (***n***_ 2_ = −***n***), and medium 1 on the positive side (***n***_ 1_ = ***n***). Taking this into account as well as the matching boundary condition for ***D***, $D_{1n}-D_{2n}=\sigma_{12}$, equation (2.5) and D=εE result for *σ*_12_ the expression

2.6 $$\sigma_{12}=\varepsilon_{2}\left[\frac{\eta_{1}}{\eta_{2}}-\frac{\varepsilon_{1}}{\varepsilon_{2}}\right]\frac{\partial \phi_{1}}{\partial n}=-\varepsilon_{2}\left[\frac{\eta_{1}}{\eta_{2}}-\frac{\varepsilon_{1}}{\varepsilon_{2}}\right]E_{1n},$$

where *D*_1*n*_ and *D*_2*n*_ are the normal components of the flux density vector ***D*** in the tumour and the surrounding healthy tissue, respectively.

### 2.5. Calculation of the free electric charge surface density *σ*_12_

Strictly speaking, the problem to be solved for the calculation of the electric potential is equation (2.3) subject to the matching boundary conditions for *ϕ* and ∂*ϕ*/∂*n*

2.7 $$\{\begin{array}{c} \nabla^{2}\phi =\nabla ∙E_{f}(r)\\ \phi_{1}=\phi_{2}\;\\ \eta_{1}\frac{\partial \phi_{1}}{\partial n}=\eta_{2}\frac{\partial \phi_{2}}{\partial n} \end{array},$$

where r∈Σ.

The tumour spherical model is reported in [41,42]. As *ϕ* at *Σ* may be experimentally measured, conditions that may be replaced by a condition of Dirichlet and the work region is only inside the spherical tumour, of radius *R*, the solution of the problem of Poisson into the tumour in spherical coordinates (*r*,*θ*,*φ*; 0 ≤ *r* < *R*, 0 ≤ *θ* ≤ π and 0 ≤ *φ* ≤ 2π) is given by

2.8 $$\phi_{1}(r,\theta ,\varphi )=\phi_{1h}+\phi_{1p}=\overset{\infty }{\underset{n=0}{\sum }}\overset{n}{\underset{m=0}{\sum }}r^{n}P_{n}^{m}(\cos\theta )(A_{nm}\cos m\varphi +B_{nm}\mathrm{sen}m\varphi )+\phi_{1p},$$

where $P_{n}^{m}(\cos\theta )$ are the generalized polynomials of Legendre and *ϕ*_1*p*_ is a particular solution any of equation (2.3) in the tumour.

Assumption 7 supposes that $\nabla ∙E_{f}=2/r$. In this case, the solution (2.8) is bounded and it does not depend on the coordinates *θ* and *φ*, given by

2.9 $$\phi_{1}=A\frac{r}{R}+A_{00}.$$

Constants *A* and *A*_00_ in equation (2.9) are calculated from *ϕ*_0_ = *ϕ*_1_(*r* = 0) and *ϕ_s_* = *ϕ*_1_(*R*, 0, 0), being $A=\phi_{s}-\phi_{0}$ and $A_{00}=\phi_{0}$. As a result, *ϕ*_1_(*r*) is given by

2.10 $$\phi_{1}(r)=\frac{\phi_{s}-\phi_{0}}{R}r+\phi_{0},\;\;\;0\leq r\leq R.$$

In equation (2.10), the difference between *ϕ*_0_ and *ϕ_s_* represents the tumour heterogeneity from the electrical point of view. The term (*ϕ*_0_-*ϕ_s_*)/*R* is interpreted as the linear radial gradient of *ϕ*_1_(*r*).

The electric field intensity in the tumour is calculated from $E_{1}=-\nabla \phi_{1}$, given by

2.11 $$E_{1}(r)=\frac{\phi_{0}-\phi_{s}}{R},\;\;\;0\leq r\leq R.$$

Equation (2.11) shows that the electric field is uniformly distributed in the entire tumour volume. If equation (2.11) is substituted in equation (2.6), the following expression is found for *σ*_12_, given by

2.12 $$\sigma_{12}=-\varepsilon_{2}\left[\frac{\eta_{1}}{\eta_{2}}-\frac{\varepsilon_{1}}{\varepsilon_{2}}\right]\left[\frac{\phi_{0}-\phi_{s}}{R}\right].$$

Equation (2.12) gives the dependence of *σ*_12_ with *ϕ*_0_, *ϕ*_s_, *R*, *η_k_* and *ε_k_* (*k* = 1,2) for a fixed time after tumour cells are inoculated in the organism. *R* is any tumour radius higher than and equal to *R*_m_, where *R*_m_ is the minimum tumour radius measured in preclinical studies or the first tumour radius detected in clinics [1,2]. The term (*η*_1_/*η*_2_−*ε*_1_/*ε*_2_) represents the difference between the conductive and dielectric ratios of the tumour and the surrounding healthy tissue.

Several experimental studies report that *R* of untreated tumours changes in time *t* [1–3]. As a result, *σ*_12_ is expected to depend on *t*. For this, CGE is used.

### 2.6. Conventional Gompertz equation

CGE is given by

2.13 $$V_{T}(t)=V_{T0}e^{\left(\alpha /\beta )(1-e^{-\beta t}\right)},$$

where *V*_T_(*t*) represents the tumour volume at a time *t* after tumour cells are inoculated into the host. The initial tumour volume (*V*_T0_) is given by the initial condition *V*(*t* = 0) = *V*_T0_. The parameter *α* (*α* > 0) is the intrinsic growth rate of the tumour. The parameter *β* (*β* > 0) is the growth deceleration factor due to the endogenous antiangiogenic process [1,2,4].

As the tumour is assumed a spheroid, *V*_T_(*t*) in CGE corresponds to the volume of a sphere ($V_{T}(t)=4\pi R_{T}^{3}(t)/3$, where *R*_T_(*t*) is the spheroid tumour radius at a time *t*). As *R*_T_(*t*) and *V*_T_(*t*) depend on *t*, *R* in equation (2.12) is replaced by *R*_T_(*t*). As a result, *σ*_12_ is a function of *t*, named *σ*_12_(*t*). Substituting *V*_T_(*t*) in equation (2.13) results in

2.14 $$R_{T}(t)=R_{T}=R_{T0}\sqrt[{3}]{e^{\left(\alpha /\beta )(1-e^{-\beta t}\right)},}$$

where *R*_T0_ satisfies the initial condition *R*_T_(*t* = 0) = *R*_T0_ (figure 1).

The substitution of equation (2.14) in equation (2.12) allows us to express approximately *σ*_12_ in terms of *R_T_*_0_, *ϕ*_0_, *ϕ*_s_, *η*_1_, *ε*_1_, *η*_2_, *ε*_2_, *i*, *i*_0_, *α*, *β* and *t*, unprecedented in the literature. In this study, three graphs for *R*_T_ (*R*_T_ versus *t*, d*R*_T_/dt versus *t*, and d*R*_T_/dt versus *R*_T_) and three graphs for *σ*_12_ (*σ*_12_ versus *R*_T_, d*σ*_12_/dt versus *t*, and d*σ*_12_/dt versus *σ*_12_) are analysed, where d*R*_T_/dt is the first derivative of *R*_T_ with regard to *t* whereas d*σ*_12_/dt is the first derivative of *σ*_12_ with respect to *t*. From these six graphs, four graphs are only shown in this study: *R*_T_ versus *t*, d*R*_T_/dt versus *R*_T_, *σ*_12_ versus *R*_T_, and d*σ*_12_/dt versus *σ*_12_.

### 2.7. Simulations

For simulations, we use values of *α* (0.6 days^−1^) and *β* (0.2 days^−1^) corresponding to the fibrosarcoma Sa-37 tumour [1,2], *R*_T0_ (5.6 mm) and *ϕ*_0_ = −160 mV corresponding to the LLC tumour [44], and different values of *ϕ*_s_ (between −15 and −135 mV) and *η*_1_/*η*_2_−*ε*_1_/*ε*_2_ (between 1 and 5). For these values of *ϕ*_0_ and *ϕ*_s_, *ϕ*_0_−*ϕ*_s_ varies between −145 and −25 mV. In this study, we only show results for *ϕ*_0_−*ϕ*_s_ (−145 and −25 mV) and *η*_1_/*η*_2_−*ε*_1_/*ε*_2_ (1, 3 and 5). Furthermore, the parameter *ε*_2_ in equation (2.12) is calculated by the expression *ε*_2_ = *ε*_r2_*ε*_0_, where *ε*_0_ (8.85 × 10^−12^ F/m) is the vacuum permittivity and *ε*_r2_ (4 × 10^7^) the relative permittivity of the muscle. Muscle is one type of tissue in which tumour cells are more frequently inoculated subcutaneously [1,2]. This is why the muscle and its electrical properties are chosen in this study to characterize the healthy tissue that surrounds the tumour.

The aforementioned range of *ϕ_s_* may be justified for the following three reasons. First, *ϕ*_s_ is unknown experimentally and theoretically. Second, *ϕ* are less negative towards the peripheries of LLC and fibrosarcoma Sa-1 tumours [44]. Third, approximate knowledge of how *σ*_12_ at *Σ* is affected by difference of *ϕ* between the centre and border of the tumour from the bioelectrical point of view. This is taken into account because the tumour is more aggressive for the greater difference between the centre and border of the tumour from an oncological point of view [39]. That is why we do not use *ϕ*_0_ = −131.5 mV (for fibrosarcoma Sa-1 tumour) [44] for simulation. Fourth, *ϕ_s_* depends on the histological variety and size of the tumour, organ/tissue where it grows, type of medium (cell culture, *ex vivo* tissue or organism (i.e. animal, body human)).

Many authors report *η*_1_, *η*_2_, *ε*_1_ and *ε*_2_ values for different tumour histological varieties [6,10–12,28,36]. We calculate *η*_1_/*η*_2_ and *ε*_1_/*ε*_2_ ratios for each tumour type and all satisfy that 0 < *η*_1_/*η*_2_−*ε*_1_/*ε*_2_ < 5. *η*_1_/*η*_2_−*ε*_1_/*ε*_2_ = 0 (*η*_1_*ε*_2_ = *ε*_1_*η*_2_) supposes that the tumour and surrounding healthy tissue have the same electrical properties, in contrast with the experiment [6,10–12,28,36]. If *η*_1_/*η*_2_−*ε*_1_/*ε*_2_ increases, the conductor properties prevail in both tissues; therefore, they behave as electrical conductors, being marked for the tumour. Contrastingly, the conductor and dielectric properties prevail in these two tissues when *η*_1_/*η*_2_−*ε*_1_/*ε*_2_ is small. In this case, both tissues behave as real dielectrics. This may be relevant in the aggressiveness and therapeutic planning of tumours [29,30]. These are the reasons why we varied *η*_1_/*η*_2_−*ε*_1_/*ε*_2_ between 1 and 5.

A computer program is implemented in the Matlab software (version R2012b 64-bit, University Institute for Research in Mathematics and Applications, University of Zaragoza, Zaragoza, Spain) to calculate and simulate the tumour radius, free electric charge surface density and their first derivate in time. These calculations are performed on a PC with an Intel(R) core processor (TM) i7–3770 at 3.40 GHz with a Windows 10 operating system. All calculations take approximately 1 min.

## 3. Results

**Figure 2.**
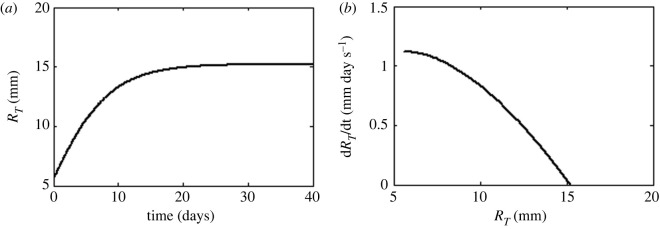
Unperturbed tumour radius. Simulations of (*a*) *R_T_* against time t, (*b*) d*R_T_*/dt versus *R_T_*.

**Figure 3.**
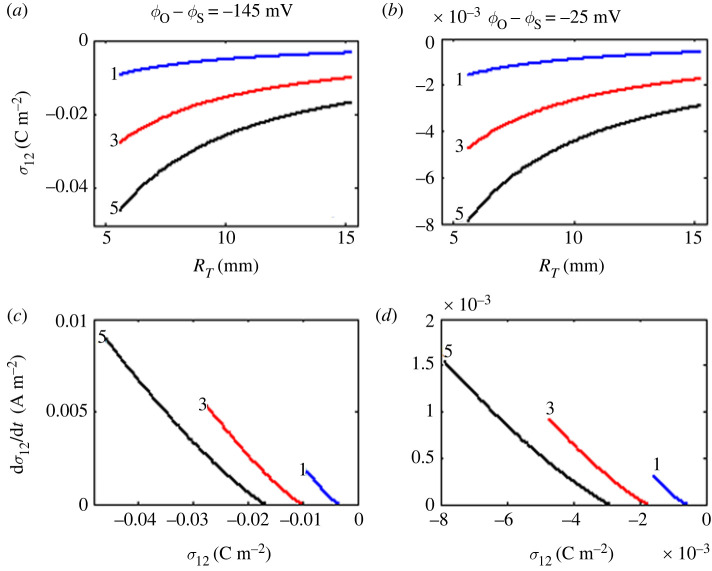
Free electric charge surface density in unperturbed tumour. Simulations of (*a*) *σ*_12_ versus *R_T_* for *ϕ*_0_−*ϕ*_s_ = −145 mV, (*b*) *σ*_12_ versus *R_T_* for *ϕ*_0_−*ϕ*_s_ = −25 mV, (*c*) d*σ*_12_/dt versus *σ*_12_ for *ϕ*_0_−*ϕ*_s_ = −145 mV, (*d*) d*σ*_12_/dt versus *σ*_12_ for *ϕ*_0_−*ϕ*_s_ = −25 mV. Three values of *η*_1_/*η*_2_−*ε*_1_/*ε*_2_ (1, 3 and 5) are shown in each sub-plot.

Figure 2 shows simulations of *R*_T_ versus *t* (figure 2*a*) and d*R*_T_/dt versus *R*_T_ (figure 2*b*). Likewise, figure 3 displays simulations of *σ*_12_ versus *R*_T_ (figure 3*a,b*) and d*σ*_12_/dt versus *σ*_12_ (figure 3*c,d*). The simulations of *σ*_12_ versus *R*_T_ and d*σ*_12_/dt versus *σ*_12_ are shown for three values of *η*_1_/*η*_2_−*ε*_1_/*ε*_2_ above-mentioned and two values of *ϕ*_0_−*ϕ*_s_ = −145 mV (figure 3*a,c*) and −25 mV (figure 3*b,d*).

The simulations of *R*_T_ versus t and *σ*_12_ versus t have similar behaviours (figure is not shown). When time elapsed, *R*_T_ and *σ*_12_ grow up to their asymptotic values reached for *t* = 40 days, called *R*_T−*f*_ and *σ*_12−*f*_, respectively. The value of *σ*_12−*f*_ (stationary condition for *σ*_12_) is less negative than *σ*_12−0_ and its value depends on *ϕ*_0_−*ϕ*_s_ and *η*_1_/*η*_2_−*ε*_1_/*ε*_2_, where *σ*_12−0_ is the value of *σ*_12_ at *t* = 0. Although the graphs of d*R*_T_/dt versus *t* and d*σ*_12_/dt versus *t* are not shown in this study, it can be proved that both graphs evidence similar behaviours. These graphics show that positive values of d*R*_T_/dt and d*σ*_12_/dt decrease asymptotically to zero when time increases. Figure 2*b* reveals that d*R_T_*/dt decreases nonlinearly to zero when *R*_T_ increases, while d*σ*_12_/dt decreases when *σ*_12_ is less negative (figure 3*c,d*). In addition, two stages are identified from the results shown in figures 2 and 3: the first grows rapidly (positive slope) and the second stationary (*R*_T_ and *σ*_12_ are constant over time).

## 4. Discussion

Although endogenous electric potentials and electrical properties of the cancer and surrounding healthy tissue may be measured [10–14,28], *σ*_12_ at *Σ* has not been experimentally measured or theoretically calculated for cancer. That is why our simulations have not been experimentally validated (main limitation of this study) nor stochastically. Although stochastic simulation models are used to describe TGK [45,46], they are black box and complex. Furthermore, random variations in stochastic models (due either to uncertainties on the parameter or to small population sizes) may influence the value of *σ*_12_ at *Σ*, but do not change its time behaviour. These aspects have made us use deterministic models in this study. And these models are feasible to describe TGK [1–4], they are also simple, easily understandable, and more appropriate for some customers. They also comprise a known set of inputs (i.e. *α*, *β*, *R_T_*_0_, *ϕ*_0_, *ϕ*_s_, *η*_1_, *η*_2_, *ε*_1_ and *ε*_2_ (or *ε*_0_ and *ε*_r2_)) which will result in an unique set of outputs (i.e. *R*_T_ and *σ*_12_). In our approach, all random variations are implicitly included in parameters of *σ*_12_ (i.e. *α*, *β*, *η*_1_, *η*_2_, *ε*_1_ and *ε*_2_).

If ‘realistic simulations' are taken into account [45,46], the simple mathematical approach, ‘nonrealistic tissue’ and tumour non-spherical geometry used in this study may represent a restraint for many researchers; nevertheless, we must be careful with this statement (see our comments in subsection 2.2). The assumptions in subsection 2.1 are reasonable and supported by experimental studies. Furthermore, the results of our formalism agree (in good approximation) with experimental and theoretical results reported in the literature (see below) and suggest other findings not considered recently. Therefore, the results of our biophysic-mathematical approach are valid for such considerations. The novelty of this study does not lie in the use of Ohm law, Poisson and conventional Gompertz equations and problem of boundary conditions between two dielectric media as these are well-known facts.

This study has two main achievements. First of all, the simple biophysic-mathematical approach proposed in this study that allows us to know an approximated theoretical expression that relates *σ*_12_ at *Σ* with tumour parameters (V_T0_, *α* and *β*), tumour electrical properties (*η*_1_ and *ε*_1_), bioelectrical potential in the tumour (*ϕ*_0_, *ϕ*_s_), electrical properties of surrounding healthy tissue (*η*_2_ and *ε*_2_), which is unprecedented in the literature. Likewise, explicit knowledge of *σ*_12_ at *Σ* with *α* and *β* allows us to relate *σ*_12_ at *Σ* in terms of Avrami exponent and impingement parameter [1]; apoptosis rate, fractal dimension of the tumour contour and fractal dimension of tumour mass [4,5], also unprecedented in the literature. Furthermore, this approach constitutes a rapid and simple method for visualizing both *R*_T_ and *σ*_12_ at *Σ* changes in time without using special software for numerical modelling. That is another reason why we prefer the analytical method. Second, researchers in cancer should take into account our results to increase the effectiveness of anti-cancer therapies, mainly chemotherapy, immunotherapy and physical therapies (i.e. electrochemical therapy, electroporation irreversible, hyperthermia, electrochemotherapy).

The results of this study confirm several findings reported in the literature and suggest others not yet revealed, such as: *σ*_12_ ≠ 0 at *Σ* is a direct consequence of equation (2.12) if (*η*_1_*ε*_2_−*η*_2_*ε*_1_) ≠ 0 and corroborates the existence of a multi-system with two different loss dielectrics in contact: the solid tumour and surrounding healthy tissue. Loss dielectric is a dielectric that has finite electrical conductivity and its induced electrical charges can move but not as freely as they would in a perfect conductor. If *σ*_12_ = 0 at *Σ* (*η*_1_*ε*_2_−*η*_2_*ε*_1_ = 0), *η*_1_ = *η*_2_ and *ε*_1_ = *ε*_2_, in contrast with the experiment [10,13,14,28–30]. Therefore, *σ*_12_ at *Σ* must be considered in cancer and its TGK.

As *σ*_12_ ≠ 0 at *Σ*, there are average current densities *J*_1_ (in the entire tumour volume *V* ($\int_{V}J_{1}dV\neq 0$) and a consequence of $\nabla ∙E_{f}\neq 0$) and J_2_ (in the surrounding healthy tissue), being |*J*_1_| > |*J*_2_| because *η*_1_ > *η*_2_ [10,11,14,36]. This corroborates that electrical properties, active bioelectricity (i.e. *ϕ*, concentrations and mobility of electrical charges) are much higher in the tumour than those in the surrounding healthy tissue [8,9,11,15]. *J*_1_ and *J*_2_ on both sides of *Σ* indicate the Maxwell-Wagner-Sillars effect occurs for the tumour-surrounding healthy tissue multi-system. Due to this effect, both free and bound surface charge densities contribute to *σ*_12_ (interfacial polarization). Therefore, the Maxwell-Wagner-Sillars effect must not be ignored in cancer. The motion of electrical charges in both biological tissues involved during the tumour growth happen in different time scales, named relaxation times (*τ*), *τ*_1_ being for the tumour (*τ*_1_ = *ε*_1_/*η*_1_ and it depends on the tumour histological variety) and *τ*_2_ for the surrounding healthy tissue (*τ*_2_ = *ε*_2_/*η*_2_ and it depends on the tissue type). These aspects may suggest that both tissues cannot be perfect conductors (*τ*_1_ and *τ*_2_ tend to zero because *η*_1_ and *η*_2_ are infinite) or perfect dielectrics (induced volume charges cannot move), corroborating that these two tissues are loss dielectrics.

$\nabla ∙E_{f}\neq 0$ considers that the tumour heterogeneity is implicit in the model, but not for the intra-tumour anisotropy. If the tumour and surrounding healthy tissue are assumed anisotropic, electrical properties of these two tissues should be replaced by their corresponding tensors. As a result, equations must be replaced by more complicated ones, the calculation procedure being cumbersome for obtaining the analytical solution of the problem (2.8). As $\nabla ∙E_{f}$ is positive through the tumour interior, negatively charged electrical sources prevail (*J*_1_ < 0), corroborating the tumour electronegativity (negative electric bio-potentials) [12–14] and the ionic and faradic currents should not be analysed separately [8]. This may indicate that positively charged carriers may be directed from the tumour towards the surrounding healthy tissue, explaining its electropositivity (*J*_2_ > 0) [13,14].

It should not be ignored that *J*_1_ may create a macroscopic magnetic field in the entire tumour and therefore an endogenous magnetic energy (per unit volume) that grows rapidly with increasing tumour size, in agreement with electric and magnetic fields (static or variable in time) associated with constant and time-varying endogenous electrical currents [8–10]. All these physical magnitudes are weak due to the breakdown of intercellular communication in the cancer [11,14,18] and theoretically corroborated here because $\nabla ∙E_{f}=2/r$ (divergence of $E_{f}$ decreases when *r* → *R_T_*). This corroborates that $E_{f}$ is weak and a weak electrical coupling between cancer cells, mainly in tumour regions near *Σ*, due to their higher electrophysiological activity in these regions, as documented in [1,4,5,7,13,14,18,33]. Migration of positively charged carriers from the tumour to the surrounding healthy tissue means that ionic bridges (strong interaction) among negatively and positively charged carriers are not formed and therefore weak interactions among cancer cells. Weak signals from biological systems are reported in [47]. If $E_{f}=0$, the tumour dies. $\nabla ∙E_{f}=2/r$ indicates that the highest electronegativity is in the tumour centre because $E_{f}$ is very intense in *r* = 0, a fact that may explain in part the endogenous central intra-tumour necrosis and migration of tumour cells towards *Σ*. For this, $E_{f}$ should be higher than or equal to the endogenous physiological electric field in tumours. Central intra-tumour necrosis explained here from the electrical point of view does not contradict explanations related to the lack of oxygen and nutrients in the central region of the tumour during its growth [8,48].

The time variation of *σ*_12_ at *Σ* corresponds to the change from the quick tumour growth phase to asymptotic phase of TGK and follows a sigmoidal behaviour in time, as TGK [1–5]. This nonlinear time behaviour of *σ*_12_ may be explained because *η*_1_ and *η*_2_ exhibit nonlinear behaviour due to biological tissues being nonlinear systems [1,4], and *η*_k_, *ε*_k_ (*k* = 1,2), *τ*_1_, *τ*_2_ and the relaxation time of the interfacial polarization (*τ*_p_) change in time [49]. Furthermore, these physical magnitudes, *ϕ*_o_, *ϕ*_s_ and (*ϕ*_o_−*ϕ*_s_) may change in time by dynamic self-regulation of *σ*_12_ at *Σ*; nevertheless, we do not explicitly know how. Therefore, we assume as constants these physical parameters in our approximation, being a limitation of our model.

This dynamic change of *σ*_12_ at *Σ* must be self-regulated during tumour growth, as TGK [1,4]. It is faster for the most undifferentiated tumours (most aggressive: greater difference of *α* with respect to *β*), strong endogenous electrical potential gradient in cancer (greater permissible difference between *ϕ*_o_ and *ϕ_s_*) and the greater difference is between ratios of electrical properties of the tumour and surrounding healthy tissue (maximum permissible value of *η*_1_/*η*_2_−*ε*_1_/*ε*_2_). This endogenous electrical potential gradient may explain the altered cancer bioelectricity (i.e. higher mobility of ions, electrons, charged molecules and cancer cells) [8,19]. The change from *σ*_12−0_ (more negative) to *σ*_12−*f*_ (less negative) at *Σ* supposes that negativity of *σ*_12_ changes dynamically over time during TGK, being marked for the highest values of *ϕ*_0_−*ϕ*_s_ and *η*_1_/*η*_2_−*ε*_1_/*ε*_2_. This finding may impact both chemical and electrical environments of the cancer cells and the solid tumour; the hypocellular gap on the tumour-host interface (responsible for the differentiation between tumour electrical properties and the surrounding healthy tissue) [50]; *τ_p_*, which depends on *η_k_* and *ε_k_* (*k* = 1,2) [31,32]; and spatio-temporal dynamic at *Σ* [51].

The tumour's electronegativity during its growth ($\nabla ∙E_{f}$ > 0) may be explained from generation of more negative charges produced by different redox processes, duplication of cancer cells (mainly in regions near *Σ*) and/or the dynamic self-regulation of negativity of *σ*_12_ at *Σ* (molecules and ions negatively charged, and electrons migrate in time from *Σ* towards the entire tumour interior until *σ*_12_ = *σ*_12−*f*_). This may suggest that dynamical alterations in cancer bioelectricity impact its growth, invasion, metastasis, maximum survival, neutralization of the attack of the immune system and resistance to anti-cancer therapies, as reported in previous studies [8,12–15,33,52–55]. This dynamic self-regulation of *σ*_12_ at *Σ*, $\nabla ∙E_{f}=2/r$, and cancer cells negatively charged [14] may suggest three aspects. First, the electrostatic repulsion among cancer cells facilitates migration, invasion and metastasis [53]. For this, electric biopotentials have to be more negative in the central region of the tumour than in its periphery, during its growth over time, as in [44], so that the entire tumour behaves like a negatively charged heterogeneous endogenous electrical shield. Second, electric field intensity of this shield changes dynamically over time. It depends on *ϕ*_0_−*ϕ*_s_, *η*_1_/*η*_2_−*ε*_1_/*ε*_2_ and dynamic change in *σ*_12_ at *Σ*, and electrostatically repels humoral and cellular components of the immune system, mainly those negatively charged (e.g. T lymphocytes, natural killer cells, among others) [14]. Consequently, the immune system does not recognize the tumour. Third, positive electrical charges migrate toward the surrounding healthy tissue, as reported for diffusion of hydrogen ions, which damage the normal tissue [50]. This migration of positively charged carriers through *Σ* may avoid that *σ*_12_ = 0 at *Σ* and weaken the electrostatic coupling among cancer cells (negatively charged) in tumour regions near *Σ* to favour their metastasis. This may explain the acidification of the tumour microenvironment, which is related to the progression, invasion, metastasis, stimulation of many immunosuppressive processes and resistance to anti-cancer therapies [8].

If *σ*_12−*f*_ were much more negative than *σ*_12−0_ at *Σ*, the tumour would behave as an isolated system because carriers of negative electrical charges would essentially concentrate at *Σ*. This interface behaves as an electrical barrier that prevents the entry and exit of different substances through it. If *σ*_12_ = 0 at *Σ*, the cellular elements of the immune system would enter the tumour interior. In both cases, the tumour would completely self-destruct, in contrast to the experiment [1,4,5]. Endogenous angiogenesis may be the emerging physiological mechanism to avoid *σ*_12_ = 0 at *Σ* and replace the mechanism for which *σ*_12_ at *Σ* changes from *σ*_12–0_ to *σ*_12−f_ as the tumour increases in size. This latter facilitates the migration of cancer cells toward the surrounding healthy tissue and the entry of nutrients into the tumour during its growth because blood is the most conductive tissue in the human body. This may justify why angiogenesis process in cancer emerges due to changes in its electrical and mechanical parameters at *Σ*, as previously reported in [1,4].

Although malignant tumours are not generally spherical [1–4,7], results of this study confirm the usefulness of the spheroidal model of a tumour to reveal intrinsic findings in its TGK, in accordance with [11,49]. If we consider that boundary condition depends on the spherical coordinates (*r*,*θ*,*φ*) in problem (2.8), *σ*_12_ would depend on (*R_T_*,*θ*,*φ*), which means that *σ*_12_ is not uniform at the entire *Σ*. Furthermore, *ϕ*_1_(*r*), E_1_(*r*) and *J*_1_(*r*) depend nonlinearly on (*r*,*θ*,*φ*). As the ellipsoidal geometry of the solid tumour is often observed in the experiment [1–4,7], the problem (2.8) has to be solved in elliptical coordinates. For a tumour arbitrary geometry, the solution of the problem (2.8) is more complex and requires numerical methods.

The results of this study show that *R_T_* and *σ*_12_ at *Σ* change in time during tumour growth for constant values of *α*, *β*, *ϕ*_0_, *ϕ_s_*, *η*_1_, *η*_2_, *ε*_1_, and *ε*_2_. These eight parameters as well as *ϕ*_1_(*r*), *E*_1_(*r*), and *J*_1_(*r*) are expected to change in time too due to biological changes in tumour growth, as necrosis (central or no), angiogenesis, among others. Nevertheless, there is no relevant experimental/theoretical information available that links these two biological findings with *σ*_12_ at *Σ*. Consequently, it is tedious to propose a biophysic-mathematical approach that involves time dependence of *α*, *β*, *ϕ*_0_, *ϕ_s_*, *η*_1_, *η*_2_, *ε*_1_, and *ε*_2_ in time changes of *R_T_* and *σ*_12_ at *Σ*. With this in mind, a longitudinal study is required to allow each of these eight parameters to be measured in time. It is important to point out that values of *ϕ*_0_, *ϕ_s_*, *η*_1_, *η*_2_, *ε*_1_, and *ε*_2_ are reported in transversal studies [6,7,10–13,29,36,37,44]; therefore, these values cannot be extrapolated to other time instants.

Tumour necrosis is caused by nutrient and oxygen deprivation, and metabolic stress. The content of necrotic cells enhances angiogenesis and proliferation of endothelial cells, induces vasculature, as well as increasing migration, invasion and cell-cell interaction. Both necrosis and angiogenesis impact directly on cancer promotion and on the tumour microenvironment, as well as on cancer resistance and recurrence [56,57]. The influence of necrosis and angiogenesis on *σ*_12_ at *Σ* may be explained from equations (2.12) and (2.14). The tumour necrosis leads to an increase of *α* parameter, whereas tumour angiogenesis brings about an increase of the parameter *α* and a decrease of the parameter *β* (1/*β* dominates the term ($1-e^{-\beta t}$) in the exponent of equation (2.14)). Consequently, *R*_T_ increases and *σ*_12_ at *Σ* decreases in absolute value (*σ*_12_ at *Σ* makes more positive) in both cases. It should be noted that decrease of *β* during tumour growth means that the balance between the productions of angiogenic and antiangiogenic molecules is dominated by angiogenic molecules.

The cell loss factors (CLFs: necrosis, apoptosis, exfoliation and metastasis) should be carefully analysed in untreated tumours. These CLFs should be small so that the doubling time of the tumour (DT) is short, according to the Steel equation ($\mathrm{DT}=T_{c}\ln2/[\left(1-CLFs)(1+GF\right)]$, where *T*_c_ and *GF* are the cell cycle average time and tumour growth factor, respectively) [58]. For instance, our vast experience in preclinical studies indicates that the tumour necrosis percentage varies between 10 and 30% of the entire tumour volume, depending on tumour histological variety, *V*_T0_, host and observation period of the study [1,2,4]. Short DT leads to an increase of *α* and decrease of *σ*_12_ at *Σ*. This may be explained by the following expression obtained by substituting $V_{T}(t=\mathrm{DT})=2V_{T0}$ in equation (2.13), given by $\alpha =\beta \ln2/(1-e^{-\beta t})$. An increase in the number of cells that participate in the cell cycle (*N*_cc_) leads to an increase of GF ($\mathrm{GF}=N_{\mathrm{cc}}/(N_{\mathrm{cc}}+N_{n-\mathrm{cc}})$), where *N_n_*_−cc_ is the number of cells that do not participate in the cell cycle. As a result the increase of GF, DT is short, *α* increases and *σ*_12_ at *Σ* decreases.

When the tumour grows it becomes more heterogeneous, as it demonstrates simulations for its spherical and non-spherical geometries [43]. The tumour heterogeneity has one of the main roles in cancer promotion and on the tumour microenvironment, as well as on cancer resistance and recurrence [8,14,39,43,56,57]. Therefore, it is considered a cancer hallmark. From the biological point of view, a more heterogeneous tumour brings about an increase of *α* and therefore a decrease of *σ*_12_ at *Σ*. This statement is corroborated from a bioelectric point of view with equation (2.12), as discussed above.

The simulations shown in [43] suggest that the spherical tumour has greatly defined its layers compared to ellipsoidal tumours, which may validate why the spherical tumour is a good model to study chemo-resistance and radio-resistance [41,42]. The tumour heterogeneity may be simulated approximately from a biophysical point of view following the same ideas of this study. Thus, we assume the spherical tumour formed by *M*_T_ concentric layers, each one of them of radius *R*_T*i*_, average electrical conductivity *η_i_* and average electrical permittivity *ε_i_*; -*ϕ*_0_ in the tumour centre; -*ϕ_si_* in the contour between two adjacent layers (*Σ_i_*_(*i* + 1)_) and it satisfies −*ϕ*_0_ < −*ϕ_s_*_1_ < ··· < −*ϕ_sN_*, keeping in mind [44]; and the existence of a surface charge density (*σ_i_*_(*i* + 1)_) at *Σ_i_*_(*i* + 1)_, such that: −*σ_i_*_(*i* + 1)_ < −*σ*_(*i* + 1)(*i* + 2)_ (*i* = 1,…, *M_T_*). Furthermore, there is a surface charge density (*σ_M_*_(*M* + 1)_) at border (*Σ_M_*_(*M* + 1)_) between the outermost layer of the tumour and surrounding healthy tissue (average electrical conductivity *η_M_*_+ 1_ and average electrical permittivity *ε_M_*_+ 1_). For this case, the result is

4.1 $$\begin{array}{cc} \sigma_{i(i+1)}=-\varepsilon_{i+1}\left[\frac{\eta_{i}}{\eta_{i+1}}-\frac{\varepsilon_{i}}{\varepsilon_{i+1}}\right]\left[\frac{\phi_{0}-\phi_{si}}{R_{i}}\right] & i=1,\ldots ,\;M_{T}. \end{array}$$

The condition −*σ_i_*_(*i* + 1)_ < −*σ*_(*i* + 1)(*i* + 2)_ (*i* = 1, … , *M_T_*) supposes that each tumour layer behaves as an electrical shield, with the innermost layer being the most negative, as discussed above. By contrast, the solid tumour is self-destructed, in contrast with the clinics [1–4]. Furthermore, the existence of −*σ_i_*_(*i* + 1)_ (*i* = 1, … , *M_T_*) may explain that the spherical tumour has well-defined multicentric layers from an electrical point of view, in agreement with well-defined multicentric layers from a biological point of view [43]. This confirms the close relationship between electrical and physiological parameters in biological tissues [10,11,14,15,36]. Nevertheless, the equation (4.1) has the inconvenience that *M_T_*, *R_Ti_*, *η_i_*, *ε_i_*, *ϕ_si_*, *σ_i_*_(*i* + 1)_ at *Σ_i_*_(*i* + 1)_, *σ_M_*_(*M* + 1)_ at *Σ_M_*_(*M* + 1)_ (*i* = 1,…, *M_T_*) are not known neither experimentally nor theoretically. The measurement of these parameters in a multilayer tumour is more cumbersome than in a simple model, as proposed in this study. That is why we do not include a tumour with different concentric layers in the simulations proposed.

The electrical properties and active bioelectricity inherent in cancer and surrounding healthy tissue, as a whole, cannot be analysed as the sum of all processes that occur at the molecular and cellular levels. This may be argued because biological systems are by nature multiscale and formed by closely interconnected and hierarchically organized multiple subsystems and supersystems, resulting in large networks of physical or functional proximities. Subsystems are referred to biological entities in the order of nanometers (i.e. amino acids residues), angstroms (i.e. single atoms), tens to hundreds of nanometers (i.e. proteins), several microns (i.e. organelles, cells). Supersystems are referred to tissues, organs and individuals measured in fractions of meters [59].

Large networks of systems in cancer patients allow us to suggest that alterations are not only due to changes at tissue, cellular and molecular levels [8], but also to nanometric changes, as reported in [60]. Furthermore, the integral characterization of cancer patients by means of an integrated analysis of clinical-biological(tumour and patient)-functional-bioelectrical parameters [61] is possible from these larger networks. The cancer fractality at submicron [60] and tissue [1,4,5] levels confirms the close relation of the multiscale hierarchies in malignant tumours.

### 4.1. Insights about cancer therapy

Many molecules used in chemotherapy and immunotherapy are positively/negatively charged and have not given a definitive solution to the cure for cancer. Our simulations indicate that anti-cancer therapies should take into account that bioelectricity cancer cells and *σ*_12_ at *Σ* are negative to reestablish the bioelectrical states and *V*_mem_ of cancer cells within the physiological range, as reported by Cervera *et al*. [9], who recommend that the use of non-physiological perturbations would not be necessary for cancer. Although the exact mechanism is poorly understood, different cancer types generate specific galvanotaxis responses to low direct current electric fields [8,62]. The results of this study confirm that anodes (positive electrodes) should be inserted in tumour regions near *Σ* to avoid metastasis of cancer cells when electrochemical therapy is used. This may be explained because anodes generate positively charged carriers (e.g. hydrogen ions) that may intensify electrostatic interactions between negatively charged carriers (e.g. cancer cells) by means of the formation of ionic bridges. Consequently, a possible anti-cancer therapy that inhibits the release of positively charged carriers from the tumour may be suggested. Furthermore, knowledge of shape and orientation of *σ*_12_ may be essential to elucidate if anodes should be inserted in regions near *Σ* with higher or smaller *σ*_12_ values to maximize tumour volume destruction with the minimum damage to the surrounding healthy tissue.

This study opens new questions that may be essential to understand TGK and how electrophysiological variables of the untreated tumour change during its growth that may be relevant for individualized anti-cancer therapies. Among the possible questions that arise: (1) Does the tumour growth bring about change from *σ*_12−0_ to *σ*_12−*f*_ at *Σ* or does this change lead to the tumour growth? (2) Do biological changes (e.g. metabolism abnormalities) lead to physical changes (e.g. changes in *V*_mem_ and electrical properties) [43] or vice versa [40]? We believe that dynamical bioelectrical changes are primary mechanisms involved in cancer that lead to chemical changes, to biological modifications, and to clinical alterations (secondary mechanisms). (3) Are the negative charged molecules crossing *σ*_12_ at *Σ* (from surrounding cancer tissue) more easily than the positive ones? (4) Do the negatively charged molecules that cross *σ*_12_ at *Σ* induce the highest antitumour effectiveness than those positively charged? A meta-analysis may be carried out to give answers to these questions and others related to them. The cancer bioelectric handling has been suggested as a useful tool to understand bioelectrical fields that change dynamically during cancer growth and possible anti-cancer therapeutic targets, aspects that remain unclear as yet [8,43]. (5) What relationship exists between *σ*_12_ and the tumour contour fractal dimension reported in [1,4,5]? (6) What implication does non-homogeneous distribution of *σ*_12_ at *Σ* have during tumour growth? (7) What expression adopts *σ*_12_ when a heterogeneous tumour and nonlinear *ϕ*_1_ are considered? (8) Can electrochemical therapy with low-level of direct current re-establish physiological bioelectrical disorders that happen in an untreated tumour? (9) How do the endogenous magnetic field and the ellipsoidal geometry influence the untreated tumour growth? (10) How does *σ*_12_ relate to other biophysical-chemical processes that occur in the tumour? (11) How does *σ*_12_ at *Σ* change experimentally over time during the growth of untreated and treated solid tumours using any experimental techniques reported in [34,35] (e.g. electrostatic force microscopy)?

On the other hand, the results of this study may contribute to answering to the fourth challenge reported in [59], related to the capture of dynamics in multiscale models because nanometric, atomic, molecular, cellular and tissue processes are highly dynamic [1,59].

## 5. Conclusion

In conclusion, results corroborate the correspondence between the electrical and physiological parameters in the untreated cancer, which may have an essential role in its growth, progression, metastasis and protection against immune system attack and anti-cancer therapies. In addition, knowledge of *σ*_12_ at *Σ* may be relevant in the redesign of chemotherapy and immunotherapy that take into account the polarity of the substances or the design of completely novel therapies.

## Data Availability

Our work is purely theoretical and all the data needed to perform the simulations appear in the manuscript under the heading ‘Simulations’.

## References

[1] González MM et al. 2017 Is cancer a pure growth curve or does it follow a kinetics of dynamical structural transformation? BMC Cancer **17**, 174. (10.1186/s12885-017-3159-y) 28270135 PMC5339962

[2] Goris NAV et al. 2020 Efficacy of direct current generated by multiple-electrode arrays on F3II mammary carcinoma: experiment and mathematical modeling. J. Transl. Med. **18**, 1-17. (10.1186/s12967-020-02352-6) 32381006 PMC7206687

[3] Marušic M. 1996 Mathematical models of tumor growth. Math. Commun. **1**, 175-192. See https://hrcak.srce.hr/file/2874.

[4] Castañeda ARS, Torres ER, Goris NAV, González MM, Reyes JB, González VGS, Schonbek M, Montijano JI, Cabrales LEB. 2019 New formulation of the Gompertz equation to describe the kinetics of untreated tumors. PLoS ONE **14**, e0224978. (10.1371/journal.pone.0224978) 31715625 PMC6850893

[5] Goris NV, Castañeda AS, Ramirez-Torres E, Reyes JB, Randez L, Cabrales LB, Montijano J. 2020 Correspondence between formulations of Avrami and Gompertz equations for untreated tumor growth kinetics. Rev. Mex. Fis. **66**, 632-636. (10.31349/RevMexFis.66.632)

[6] Bera TK. 2018 Bioelectrical impedance and the frequency dependent current conduction through biological tissues: a short review. IOP Conf. Ser.: Mater. Sci. Eng. **331**, 012005. (10.1088/1757-899x/331/1/012005)

[7] Serša I, Beravs K, Dodd NJF, Zhao S, Miklavčič D, Demsar F. 1997 Electric current density imaging of mice tumors. Magn. Reson. Med. **37**, 404-409. (10.1002/mrm.1910370318) 9055231

[8] Robinson AJ, Jain A, Sherman HG, Hague RJM, Rahman R, Sanjuan-Alberte P, Rawson FJ. 2021 Toward hijacking bioelectricity in cancer to develop new bioelectronic medicine. Adv. Ther. **4**, 2000248. (10.1002/adtp.202000248)

[9] Cervera J, Pietak A, Levin M, Mafe S. 2018 Bioelectrical coupling in multicellular domains regulated by gap junctions: a conceptual approach. Bioelectrochemistry **123**, 45-61. (10.1016/j.bioelechem.2018.04.013) 29723806

[10] Foster KR, Schwan HP. 1996 Dielectric properties of tissues. In Handbook of biological effects of electromagnetic fields (eds C Polk, E Postow), pp. 68-70. Boca Raton, FL: CRC Press LLC.

[11] Shimonov G, Koren A, Sivek G, Socher E. 2018 Electromagnetic property characterization of biological tissues at D-band. IEEE Trans. Terahertz Sci. Technol. **8**, 155-160. (10.1109/TTHZ.2018.2789357)

[12] Hassan AM, El-Shenawee M. 2010 Modeling Biopotential signals and current densities of multiple breast cancerous cells. IEEE Trans. Biomed. Eng. **57**, 2099-2106. (10.1109/TBME.2010.2049575) 20460196

[13] Habal M, Schauble M. 1967 Electropotential differentiation of normal and tumor tissue. In Surg forum, pp. 88-90.

[14] Haltiwanger MDS. 2008 The electrical properties of cancer cells. See http://www.royalrife.com/haltiwanger1.pdf [Last update: April 2nd 2008].

[15] Wang Y, Han X, Cui Z, Shi D. 2019 Bioelectricity, its fundamentals, characterization methodology, and applications in nano-bioprobing and cancer diagnosis. Adv. Biosyst. **3**, 1900101. (10.1002/adbi.201900101) 32648718

[16] Wang Y et al. 2021 Correlation between electrical characteristics and biomarkers in breast cancer cells. Sci. Rep. **11**, 14294. (10.1038/s41598-021-93793-6) 34253828 PMC8275571

[17] Burgos-Panadero R, Lucantoni F, Gamero-Sandemetrio E, Cruz-Merino L, Álvaro T, Noguera R. 2019 The tumour microenvironment as an integrated framework to understand cancer biology. Cancer Lett. **461**, 112-122. (10.1016/j.canlet.2019.07.010) 31325528

[18] Chernet BT, Fields C, Levin M. 2015 Long-range gap junctional signaling controls oncogene-mediated tumorigenesis in Xenopus laevis embryos. Front. Physiol. **5**, 519. (10.3389/fphys.2014.00519) 25646081 PMC4298169

[19] Iorio J, Petroni G, Duranti C, Lastraioli E. 2019 Potassium and sodium channels and the Warburg effect: biophysical regulation of cancer metabolism. Bioelectricity **1**, 188-200. (10.1089/bioe.2019.0017) 34471821 PMC8370285

[20] Payne SL, Levin M, Oudin MJ. 2019 Bioelectric control of metastasis in solid tumors. Bioelectricity **1**, 114-130. (10.1089/bioe.2019.0013) 32292893 PMC6768203

[21] Bhavsar MB, Leppik L, Oliveira KMC, Barker JH. 2020 Role of bioelectricity during cell proliferation in different cell types. Front. Bioeng. Biotechnol. **8**, 603. (10.3389/fbioe.2020.00603) 32714900 PMC7343900

[22] Fan JJ, Huang X. 2020 Ion channels in cancer: Orchestrators of electrical signaling and cellular crosstalk. In Reviews of physiology, biochemistry and pharmacology, pp. 1-31. Berlin, Germany: Springer. 10.1007/112_2020_48 32894333

[23] Levin M, Martyniuk CJ. 2018 The bioelectric code: an ancient computational medium for dynamic control of growth and form. Biosystems **164**, 76-93. (10.1016/j.biosystems.2017.08.009) 28855098 PMC10464596

[24] Yang Q, Jiang N, Xu H, Zhang Y, Xiong C, Huang J. 2021 Integration of electrotaxis and durotaxis in cancer cells: Subtle nonlinear responses to electromechanical coupling cues. Biosens. Bioelectron. **186**, 113289. (10.1016/j.bios.2021.113289) 33975207

[25] Pietak A, Levin M. 2018 Bioelectrical control of positional information in development and regeneration: a review of conceptual and computational advances. Prog. Biophys. Mol. Biol. **137**, 52-68. (10.1016/j.pbiomolbio.2018.03.008) 29626560 PMC10464501

[26] Seyfried TN, Chinopoulos C. 2021 Can the mitochondrial metabolic theory explain better the origin and management of cancer than can the somatic mutation theory? Metabolites **11**, 572. (10.3390/metabo11090572) 34564387 PMC8467939

[27] Landau LD, Lifshitz EM. 1984 Electrodynamics of continuous media. In Course of theoretical physics, 3rd edn. New York, NY: Pergamon Press.

[28] Kremer F, Schönhals A. 2003 Broadband dielectric spectroscopy, 1st edn. Berlin, Germany: Springer.

[29] Sekino M, Ohsaki H, Yamaguchi-Sekino S, Iriguchi N, Ueno S. 2009 Low-frequency conductivity tensor of rat brain tissues inferred from diffusion MRI. Bioelectromagnetics **30**, 489-499. (10.1002/bem.20505) 19437459

[30] Oria EJR, Cabrales LEB, Reyes JB. 2019 Analytical solution of the bioheat equation for thermal response induced by any electrode array in anisotropic tissues with arbitrary shapes containing multiple-tumor nodules. Rev. Mex. Fis. **65**, 284-290. (10.31349/revmexfis.65.284)

[31] Das S, Gupta N. 2014 Interfacial charge behaviour at dielectric-dielectric interfaces. IEEE Trans. Dielectr. Electr. Insul. **21**, 1302-1311. (10.1109/TDEI.2014.6832278)

[32] Rogti F, Ferhat M. 2014 Maxwell–Wagner polarization and interfacial charge at the multi-layers of thermoplastic polymers. J. Electrost. **72**, 91-97. (10.1016/j.elstat.2013.11.012)

[33] Huang YJ, Hoffmann G, Wheeler B, Schiapparelli P, Quinones-Hinojosa A, Searson P. 2016 Cellular microenvironment modulates the galvanotaxis of brain tumor initiating cells. Sci. Rep. **6**, 21583. (10.1038/srep21583) 26898606 PMC4761929

[34] Fabregas R, Gomila G. 2020 Dielectric nanotomography based on electrostatic force microscopy: a numerical analysis. J. Appl. Phys. **127**, 024301. (10.1063/1.5122984)

[35] Casuso I, Redondo-Morata L, Rico F. 2020 Biological physics by high-speed atomic force microscopy. Phil. Trans. R. Soc. A **378**, 20190604. (10.1098/rsta.2019.0604) 33100165 PMC7661283

[36] Yu X, Sun Y, Cai K, Yu H, Zhou D, Lu D, Xin SX. 2020 Dielectric properties of normal and metastatic lymph nodes ex vivo from lung cancer surgeries. Bioelectromagnetics **41**, 148-155. (10.1002/bem.22246) 31912926

[37] Shawki MM, Azmy MM, Salama M, Shawki S. 2021 Mathematical and deep learning analysis based on tissue dielectric properties at low frequencies predict outcome in human breast cancer. Technol. Health Care **30**, 633-645. (10.3233/THC-213096) 34366303

[38] Silver BB, Nelson CM. 2018 The bioelectric code: reprogramming cancer and aging from the interface of mechanical and chemical microenvironments. Front. Cell Dev. Biol. **6**, 21. (10.3389/fcell.2018.00021) 29560350 PMC5845671

[39] Cotran RS, Kumar V, Collins T. 1999 Patología estructural y funcional, 6th edn, pp. 277-347. S.A.U. Madrid: McGraw-Hill Interamericana de España.

[40] Limkin EJ, Reuzé S, Carré A, Sun R, Schernberg A, Alexis A, Deutsch E, Ferté C, Robert C. 2019 The complexity of tumor shape, spiculatedness, correlates with tumor radiomic shape features. Sci. Rep. **9**, 4329. (10.1038/s41598-019-40437-5) 30867443 PMC6416263

[41] Gilazieva Z, Ponomarev A, Rutland C, Rizvanov A, Solovyeva V. 2020 Promising applications of tumor spheroids and organoids for personalized medicine. Cancers **12**, 2727. (10.3390/cancers12102727) 32977530 PMC7598156

[42] Zhang C, Yang Z, Dong DL, Jang TS, Knowles JC, Kim HW, Jin GZ, Xuan Y. 2020 3D culture technologies of cancer stem cells: promising ex vivo tumor models. J. Tissue Eng. **11**, 2041731420933407. (10.1177/2041731420933407) 32637062 PMC7318804

[43] Castañeda ARS, Del Pozo JM, Ramirez-Torres EE, Oria EJR, Vaillant SB, Montijano JI, Cabrales LEB. 2023 Spatio temporal dynamics of direct current in treated anisotropic tumors. Math. Compt. Simul. **203**, 609-632. (10.1016/j.matcom.2022.07.004)

[44] Miklavčič D, Serša G, Novaković S, Rebersek S. 1990 Tumor bioelectric potential and its possible exploitation for tumor growth retardation. J. Bioelectricity **9**, 133-149. (10.3109/15368379009119801)

[45] Ferrante L, Bompadre S, Possati L, Leone L. 2000 Parameter estimation in a Gompertzian stochastic model for tumor growth. Biometrics **56**, 1076-1081. (10.1111/j.0006-341x.2000.01076.X) 11129463

[46] Liu X, Li Q, Pan J. 2018 A deterministic and stochastic model for the system dynamics of tumor-immune responses to chemotherapy. Physica A. **500**, 162-176. (10.1016/j.physa.2018.02.118)

[47] Huang D, Yang J, Zhou D, Sanjuán MAF, Liu H. 2019 Recovering an unknown signal completely submerged in strong noise by a new stochastic resonance method. Commun. Nonlinear Sci. Numer. Simul. **66**, 156-166. (10.1016/j.cnsns.2018.06.011)

[48] Mateus R, Fuhrmann JF, Dye NA. 2021 Growth across scales: dynamic signaling impacts tissue size and shape. Curr. Opin. Cell Biol. **73**, 50-57. (10.1016/j.ceb.2021.05.002) 34182209

[49] Vu TTN, Teyssedre G, Roy SL, Laurent C. 2017 Maxwell–Wagner effect in multi-layered dielectrics: interfacial charge measurement and modelling. Technologies **5**, 27. (10.3390/technologies5020027)

[50] Enderling H, Chaplain MAJ. 2014 Mathematical modeling of tumor growth and treatment. Curr. Pharm. Des. **20**, 4934-4940. 24283955 10.2174/1381612819666131125150434

[51] Tsai F-C, Wang M-C, Lo J-F, Chou C-M, Lin Y-L. 2012 Spatiotemporal dynamics of the biological interface between cancer and the microenvironment: a fractal anomalous diffusion model with microenvironment plasticity. Theor. Biol. Med. Model. **9**, 36. (10.1186/1742-4682-9-36) 22889191 PMC3462694

[52] Saez P. 2016 On the theories and numerics of continuum models for adaptation processes in biological tissues. Arch. Comput. Methods Eng. **23**, 301-322. (10.1007/s11831-014-9142-8)

[53] Funk RHW. 2015 Endogenous electric fields as guiding cue for cell migration. Front. Physiol. **6**, 143. (10.3389/fphys.2015.00143) 26029113 PMC4429568

[54] Rianna C, Radmacher M. 2017 Influence of microenvironment topography and stiffness on the mechanics and motility of normal and cancer renal cells. Nanoscale **9**, 11 222-11 230. (10.1039/C7NR02940C) 28752168

[55] Levin M, Pezzulo G, Finkelstein JM. 2017 Endogenous bioelectric signaling networks: exploiting voltage gradients for control of growth and form. Annu. Rev. Biomed. Eng. **19**, 353-387. (10.1146/annurev-bioeng-071114-040647) 28633567 PMC10478168

[56] Karsch-Bluman A, Feiglin A, Arbib E, Stern T, Shoval H, Schwob O, Berger M, Benny O. 2019 Tissue necrosis and its role in cancer progression. Oncogene **38**, 1920-1935. (10.1038/s41388-018-0555-y) 30390074

[57] LaRue KE, Khalil M, Freyer JP. 2004 Microenvironment regulation of proliferation in multicellular spheroids is mediated through differential expression of cyclin-dependent kinase inhibitors. Cancer Res. **64**, 1621-1631. (10.1158/0008-5472.CAN-2902-2) 14996720

[58] Steel GG. 1997 The growth rate of tumours. In Basic clinical radiobiology (ed. GG Steel), pp. 8-13, 2nd edn. New York, NY: Oxford University Press.

[59] Schaffer LV, Ideer T. 2021 Mapping the multiscale structure of biological systems. Cell Syst. **12**, 622-635. (10.1016/j.cels.2021.05.012) 34139169 PMC8245186

[60] Das N, Alexandrov S, Gilligan KE, Dwyer RM, Saager RB, Ghosh N, Leahy M. 2021 Characterization of nanosensitive multifractality in submicron scale tissue morphology and its alteration in tumor progression. J. Biomed. Opt. **26**, 016003. (10.1117/1.JBO.26.1.016003) 33432788 PMC7797786

[61] Regüeiferos JCC et al. 2022 Integrated analysis of clinical, bioelectrical and functional variables in newly diagnosed lung cancer adult patients: pilot study. Transl. Med. Commun. **7**, 1-16. (10.1186/s41231-022-00127-3)

[62] Huang C-W, Cheng J-Y, Yen M-H, Young T-H. 2009 Electrotaxis of lung cancer cells in a multiple-electric-field chip. Biosens. Bioelectron. **24**, 3510-3516. (10.1016/j.bios.2009.05.001) 19497728

